# Anisodamine (654-1/654-2) ameliorates septic kidney injury in rats by inhibiting inflammation and apoptosis

**DOI:** 10.3389/fphar.2024.1421551

**Published:** 2024-09-27

**Authors:** Dong Liu, Fei Tang, Li Zhang, Feng Wan, Li-Yue Xu, Jing-Nan Zhang, Xiao-Lan Zhao, Hui Ao, Cheng Peng

**Affiliations:** ^1^ State Key Laboratory of Southwestern Chinese Medicine Resources, Chengdu University of Traditional Chinese Medicine, Chengdu, China; ^2^ Chengdu No. 1 Pharmaceutical Co. Ltd., Pengzhou, Sichuan, China; ^3^ Innovative Institute of Chinese Medicine and Pharmacy, Chengdu University of Traditional Chinese Medicine, Chengdu, China

**Keywords:** septic shock, anisodamine (654-1/654-2), acute kidney injury, apoptosis, inflammatory

## Abstract

**Introduction:**

To investigate the protective effects of anisodamine (654-1/654-2) against acute kidney injury (AKI) in LPS-induced septic shock rats and explore its molecular mechanisms.

**Methods:**

56 rats were randomly divided into 8 groups: control, LPS, LPS + 654-1, and LPS + 654-2 (1.25, 2.5 and 5 mg/kg). The model was evaluated by monitoring MAP, HR, and plasma LD levels. ELISA and biochemical assay kits were used to measure the levels of inflammatory cytokines (IL-1β, IL-6, and TNF-α) and kidney injury markers (BUN and CRE). Additionally, RNA-seq and bioinformatic analysis were performed to explore the mechanism of action of 654-1/654-2, and verification was conducted by western blotting and RT-PCR.

**Results:**

654-1/654-2 significantly restored the levels of MAP, HR, and plasma LD in septic shock rats. Furthermore, 654-1/654-2 (5 mg/kg) effectively ameliorated LPS-induced kidney structural damage and exhibited a dose-dependent reduction in levels of inflammatory cytokines and kidney injury markers. In addition, RNA-seq, WB, and RT-PCR analyses revealed that 654-1/654-2 exerted its effects by inhibiting the expressions of the NF-κB and MAPK pathways and activating the Pi3K/Akt/Bcl-2 signaling pathway, thereby mitigating AKI.

**Discussion:**

This study suggested that 654-1/654-2 could alleviate AKI in septic shock rats by improving inflammation invasion and cell apoptosis. Notably, 654-1/654-2 collectively suppressed inflammation response through the p38/JNK/AP-1/NF-κB pathway. Additionally, 654-1 promotes survival signaling via the Pi3K/Akt/Bcl-2 pathway, whereas 654-2 reduces apoptosis through the P53/Bax pathway. These findings provided a theoretical basis for the clinical application of 654-1/654-2 in treating organ damage caused by septic shock.

## Graphical Abstract

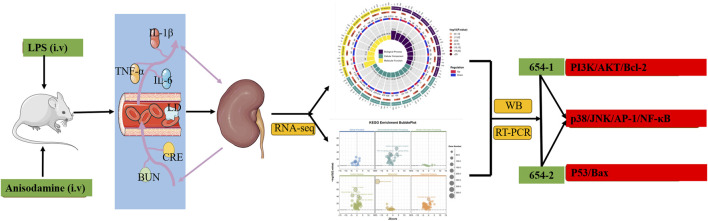

## 1 Introduction

Sepsis is a complex disease characterized by an aberrant host response to infection, resulting in acute organ dysfunction and a substantial increase in mortality rate. Septic shock is considered as a subset of sepsis (Cecconi et al., 2018). Acute kidney injury (AKI) is the most prevalent organ dysfunction among patients with septic shock. Both inflammation and cellular apoptosis play pivotal roles in the pathogenesis of septic acute kidney injury (AKI) (Morrell et al., 2014; Long et al., 2024). In lipopolysaccharide (LPS)-induced AKI mice, fisetin alleviates renal inflammation through the modulation of the NF-κB p65 and MAPK signaling pathways (Ren et al., 2020; Zheng et al., 2024). Simultaneously, activation of the Pi3K/Akt pathway is involved in the regulation of cellular apoptosis (Ullah et al., 2020; Hu et al., 2023). Additionally, sepsis-induced myocardial apoptosis was markedly attenuated by LmiR-125b transfection by suppressing p53 and Bax expression (Ma et al., 2016). Present strategies primarily concentrate on halting the progression of renal function decline. However, there is currently no authorized pharmacological intervention available for the prevention, treatment, or promotion of recovery in AKI.

Extensive research has demonstrated that numerous herb medicines possess multiple active ingredients exhibiting diverse biological activities, including anti-inflammatory, antioxidant, and anti-tumor effects. These properties have shown promising results in ameliorating refractory diseases prevalent (Liu et al., 2018; Gou et al., 2023; Liu et al., 2023; Xia et al., 2022; Wan et al., 2022; Wu et al., 2022; Yang et al., 2022; Wan et al., 2021). Anisodamine is a biogenic alkaloid extracted from the Solanaceae plant Scopolia tangutica. Currently, there are two types of anisodamine available: the natural product (654-1), which is a raceanisodamine, and the synthesized form (654-2), consisting of two enantiomers (Zhang et al., 2023). As an M-choline receptor blocker, anisodamine can be used to treat organophosphorus poisoning and microcirculatory disorders. In China, anisodamine has also been used for septic shock patients (Zhang et al., 2023). Studies have shown that anisodamine could significantly improve LPS-induced septic AKI in rats by reducing serum TNF-α, IL-6, IL-1β, and lactate (LD) levels (Wan et al., 2020). Additionally, it has been found that anisodamine inhibited serum creatinine (CRE) and blood urea nitrogen (BUN) levels and suppressed renal cell apoptosis in AKI rats (Li et al., 2019).

However, the molecular mechanisms underlying the beneficial effects of anisodamine (654-1/654-2) against septic AKI have not been thoroughly investigated to date and require further research. Additionally, the mechanisms of action between 654-1 and 654-2 remain to be studied. Therefore, the objective of this study is to examine the protective effects of 654-1/654-2 against septic AKI and explain the differences in their mechanisms. A rat model of septic shock induced by LPS was established to evaluate the success of the model by assessing mean arterial pressure (MAP) and serum lactate (LD) levels, as well as to confirm the occurrence of renal injury through serum BUN and CRE levels. To investigate the potential mechanisms of action and their differences, renal RNA-seq techniques were employed. Finally, the potential mechanisms of action were further validated using western blot and real-time quantitative RT-PCR analysis.

## 2 Materials and methods

### 2.1 Animal model

Male Sprague-Dawley rats (220–250 g) were sourced from CHENGDU DOSSY Experimental Animals CO., LTD (Chengdu, China, SCXK 2020-030). The rats were housed in a temperature-controlled facility with a constant temperature of 25°C ± 2°C and humidity of 55% ± 10%. The facility followed a 12:12-h light/dark cycle, and the rats were kept in solid-bottom cages. During the study, the rats had unrestricted access to food and water, except a 1-h fasting period before the experiments. Based on preliminary experiment results, 56 male rats were randomly divided into 8 groups: control group, LPS group, LPS + 654-1 group (high, medium, and low doses), and LPS + 654-2 group (high, medium, and low doses). Except for the control group, rats in the other groups received intravenous tail vein injections of LPS from *Escherichia coli* O55:B5 (Sigma, United States) (5 mg/kg). The treatment groups received intravenous injections of 654-1 (Chengdu NO.1 Pharmaceutical Co., Ltd., China) and 654-2 (MINSHENG Pharmaceutical Group Ltd., Co., China) at doses of 5, 2.5, and 1.25 mg/kg, respectively. The control and model groups received equivalent volumes of normal saline injection. The injections of 654-1/654-2 were administered at 1, 3, and 5 h after LPS administration. 6 h after LPS injection, blood samples were collected from the abdominal aorta, and the kidneys were harvested for further analysis. Plasma was obtained after the blood samples were centrifuged at 3,000 *g* for 15 min at 4°C. A portion of the kidney tissue was fixed in a 4% paraformaldehyde solution for histopathological evaluation, and the remaining tissues were rapidly frozen in liquid nitrogen and stored at −80°C.

### 2.2 Hemodynamics

The mean arterial pressure (MAP) and heart rate (HR) of the Con, LPS, 654-1 (5 mg/kg), and 654-2 (5 mg/kg) were assessed. Blood pressure and heart rate were monitored using the BP-600 A non-invasive blood pressure monitoring system (TECHMAN, Chengdu, China) at 0, 1, 3, 5, and 6 h after LPS administration. The blood pressure decreased rate and heart rate increase rate at 1, 3, 5, and 6 h were obtained by comparing the MAP and heart rate of each rat to the baseline values at 0 h.

MAP was calculated using the formula: MAP = diastolic BP + 1/3 × (systolic BP - diastolic BP).

### 2.3 Pathological staining

The renal tissues underwent dehydration, followed by embedding in paraffin and subsequent sectioning to a thickness of 3 μm. Hematoxylin and eosin staining were employed to facilitate histopathological evaluation of the sections. Digital images of the stained sections were obtained using a slide scanning system (Olympus Optical Co., Ltd., Tokyo, Japan).

### 2.4 Immunohistochemistry and TUNEL staining

Immunohistochemistry is used to assess the expression of the macrophage marker F4/80 (rat anti-F4/80; 1:1,000 dilution; Servicebio; Lot: GB113373) in the kidney. Terminal deoxynucleotidyl transferase-mediated dUTP nick-end labeling (TUNEL) staining was performed using the TUNEL BrightGreen Apoptosis Detection Kit (Vazyme Biotech Co., Ltd., Nanjing, China) following the manufacturer’s instructions. The TUNEL assay allowed for the visualization of apoptotic nuclei, which were stained with green fluorescein, while all kidney nuclei were counterstained with DAPI. The rate of apoptosis was determined by calculating the ratio of TUNEL-positive nuclei to DAPI-stained nuclei. The acquired data were analyzed using ImageJ software. Three random fields were selected from each sliced section, and the integrated optical density (IOD) was calculated. Microscopy with a digital camera (Nikon, Japan) was employed to capture the images.

### 2.5 Assessment of inflammation and renal dysfunction

Plasma levels of TNF-α (Lot: 9680019151122, ABclonal, Wuhan, China), IL-1β (Lot: 9680017191022, ABclonal, Wuhan, China), and IL-6 (Lot: 9680015140622, ABclonal, Wuhan, China) were measured using commercially available enzyme-linked immunosorbent assay (ELISA) kits, specific for inflammatory cytokines. Additionally, plasma levels of blood urea nitrogen (BUN) (Lot: 20221026, Nanjing, China), creatinine (CRE) (Lot: 20221026, Nanjing, China), and lactate (LD) (Lot: 20221026, Nanjing, China) were determined using biochemical assay kits to assess renal function. All measurements were analyzed using the ReadMax 1,500 microplate reader (Flash, Shanghai, China).

### 2.6 RNA extraction, library construction, and sequencing

The kidney tissue samples were obtained from rats in the control, LPS, 654-1 (5 mg/kg), and 654-2 (5 mg/kg) groups. Total RNA was extracted and purified using TRIzol reagent (Invitrogen, Carlsbad, CA, United States) according to the manufacturer’s protocol. The concentration and purity of the RNA samples were determined using a NanoDrop ND-1000 spectrophotometer (NanoDrop, Wilmington, DE, United States). The integrity of the RNA was assessed using a Bioanalyzer 2100 (Agilent, CA, United States), with a RIN (RNA Integrity Number) > 7.0, and confirmed by electrophoresis on denaturing agarose gel. Following sequencing, the expression levels of mRNAs were quantified using stringTie by calculating Fragments Per Kilobase of transcript per Million mapped reads (FPKM). Differentially expressed mRNAs were identified based on a fold change >2 or <0.5, and statistical significance (*P* < 0.05) using the parametric F-test based on nested linear models, performed using the edgeR package in R. To further analyze the genes, Gene Ontology (GO) and Kyoto Encyclopedia of Genes and Genomes (KEGG) enrichment analysis were performed using the DAVID software (https://david.ncifcrf.gov/).

### 2.7 Real-time quantitative RT-PCR

The kidney tissue samples were obtained from rats in the Con, LPS, 654-1 (5 mg/kg), and 654-2 (5 mg/kg) treatment groups. Total RNA was extracted from the rat kidney tissues using the Foregene total RNA isolation kit (FOREGENE, China). The extracted RNA was then reverse-transcribed into complementary DNA (cDNA) using the RT Easy™ II kit (with gDNase) (FOREGENE, China). Quantitative reverse transcription polymerase chain reaction (qRT-PCR) was performed using the Real-Time PCR Easy™-SYBR Green I kit (FOREGENE, China) and the Archimed X4 System (ROCGENE, China). The primer sequences used in the experiments are provided in Table 1. The expression levels of the target mRNAs were normalized to the expression of the reference gene GAPDH and calculated using the 2^−ΔΔCt^ method.

**TABLE 1:** Primer sequences used in this experiment.

Primer	Forward sequence	Reverse sequence
Cdkn1a	TGT​GAT​ATG​TAC​CAG​CCA​CAG​G	GGC​TCA​GGT​AGA​TCT​TGG​GC
Mapk12	TTG​GCT​CTG​GTG​CCT​ATG​GTG	CCG​GCA​GCG​TGG​ATA​TAC​TTC
Mapk10	CCA​TCG​AAG​AAT​GGA​AAG​AAC​TC	GAG​GGC​TGG​CCT​TTG​ACT​AC
Rela	CGA​TGC​ATC​CAC​AGC​TTC​CAG	TAA​TGG​CTT​GCT​CCA​GGT​CTC
Pik3r3	CTT​GTT​CTG​TGG​TTG​CAG​ACG	GTG​ACG​TTG​AGG​GAG​TCG​TT
Bcl2	TCA​TGT​GTG​TGG​AGA​GCG​TC	AGT​TCC​ACA​AAG​GCA​TCC​CAG
Nfkbie	ACA​ACC​TTT​ACC​AGA​CAG​CG	TTC​CTC​TGC​AAT​GTG​GCG​AT
Junb	AAG​ACC​AGG​AGC​GCA​TCA​AA	TTG​ACC​CCT​AGC​AGC​AAC​TG
AKT1	GAG​ACG​ATG​GAC​TTC​CGG​TC	ACT​CGT​TCA​TGG​TCA​CAC​GG
Fos	CTC​TGA​CTC​ACT​GAG​CTC​GC	CAC​AGC​CTG​GTG​TGT​TTC​AC
TP53	CAG​CAC​ATG​ACG​GAG​GTT​GT	TCA​TCC​AAA​TAC​TCC​ACA​CGC
Bax	CGG​CGA​ATT​GGA​GAT​GAA​CTG	AGC​AAA​GTA​GAA​GAG​GGC​AAC​C
GAPDH	GAA​GGT​CGG​TGT​GAA​CGG​AT	CCC​ATT​TGA​TGT​TAG​CGG​GAT

### 2.8 Western-blot analysis

Kidney tissue samples were obtained from rats in the Con, LPS, 654-1 (5 mg/kg), and 654-2 (5 mg/kg) treatment groups. The kidney tissue samples were homogenized using a glass homogenizer and then lysed with RIPA lysis solution containing PMSF. The resulting supernatant was collected using a BCA kit from Oriscience (China) to determine the protein concentration. Based on the molecular weight of the target protein, an SDS-PAGE gel was prepared, and electrophoresis was performed. The proteins were then transferred onto polyvinylidene difluoride (PVDF) membranes (0.45 μm, Millipore, Billerica, MA, United States). The membranes were subsequently blocked with 5% BSA at room temperature for 2 h and incubated overnight at 4°C with primary antibodies. The primary antibodies used, including P65, p-IκBα, IκBα, Pi3k, Akt, p-Pi3k, p-Akt, p38, caspase-3, cleaved caspase-3, Bax, Bcl-2, P53 and the loading control antibody β-actin levels were performed using specific primary antibodies as below: rabbit anti-P65, (1:2000 dilution; affinity; Lot: #52k0031), rabbit anti-p-IκBα, (1:2000 dilution; affinity; Lot: #47y8501), rabbit anti-IκBα, (1:2000 dilution; affinity; Lot: #60k0141), rabbit anti-Pi3K, (1:2000 dilution; affinity; Lot: #54f8512), rabbit anti- Akt, (1:2000 dilution; affinity; Lot: #34d5362), rabbit anti-p-Pi3K, (1:2000 dilution; affinity; Lot: #51u8853), rabbit anti-p-Akt, (1:2000 dilution; affinity; Lot: #48k0931), rabbit anti-p38, (1:2000 dilution; affinity; Lot: #76k0874), rabbit anti-caspase-3, (1:2000 dilution; affinity; Lot: #76i4559), rabbit anti-cleaved caspase-3, (1:2000 dilution; affinity; Lot: #15z0096), rabbit anti-P53, (1:2000 dilution; affinity; Lot: #31k0077), rabbit anti-Bax, (1:2000 dilution; affinity; Lot: #44q6915), rabbit anti-Bcl-2, (1:2000 dilution; affinity; Lot: #11o9905), rabbit anti-β-actin, (1:8000 dilution; Bioss; Lot: bs-0745R). After primary antibody incubation, the membranes were washed and probed with secondary antibodies (bs-40295G-HRP Bioss, China). Chemiluminescent signals were generated using an ECL kit from Oriscience (China). All immunoblot quantifications were performed in triplicate. Densitometry analysis was carried out using ImageJ software.

### 2.9 Statistical analysis

Statistical analysis was conducted using GraphPad Prism 9 software. If the data followed a normal distribution, a one-way analysis of variance (ANOVA) was used for group comparisons. Alternatively, for data that did not follow a normal distribution, the Kruskal-Wallis test followed by the Dunn’s test was employed. A significance level of *P* < 0.05 was considered statistically significant.

## 3 Results

### 3.1 654-1/654-2 improved hemodynamics in septic shock rats

**FIGURE 1:**
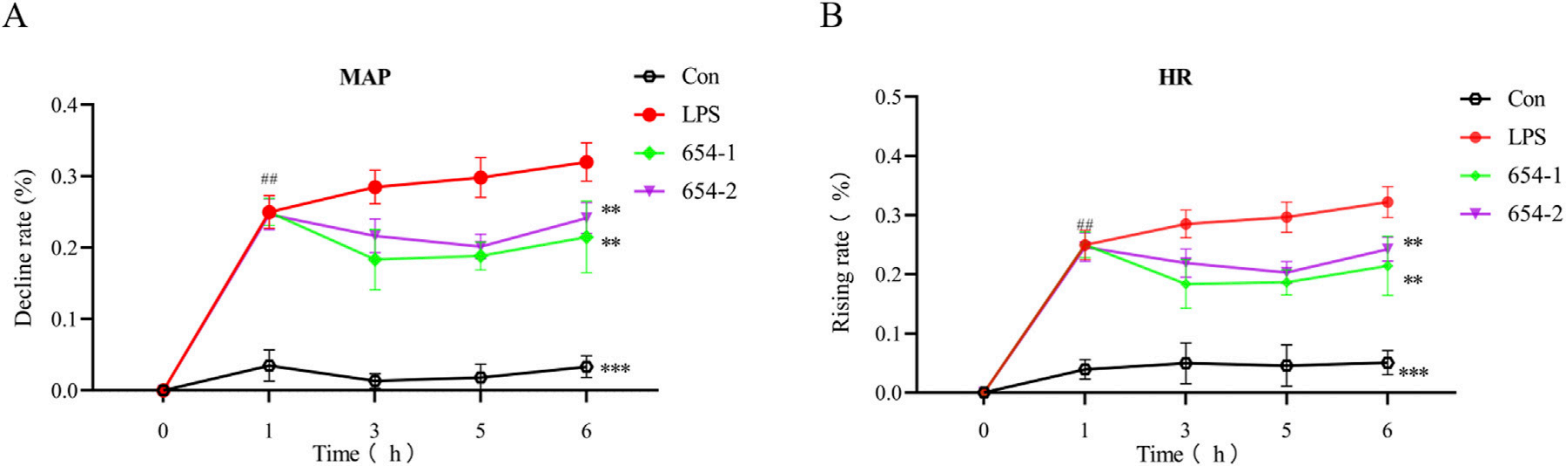
654-1/654-2 (5 mg/kg) improved the hemodynamic parameters in LPS-induced septic shock rats (n = 7). **(A)** mean atrial pressure (MAP); **(B)** heart rate (HR). Mean ± SD, **P < 0.01, ***P < 0.001 as compared to the LPS group.

After 1 h, compared to the Con group, the LPS group, as well as the groups treated with 654-1 (5 mg/kg) and 654-2 (5 mg/kg), showed a decrease of approximately 30% in mean arterial pressure (MAP) and an increase of approximately 30% in heart rate (HR), indicating successful modeling. After 6 h, compared to the Con group, the LPS group exhibited a further decrease in MAP (Figure 1A) and a further increase in HR (Figure 1B) (*P* < 0.001). In contrast, when compared to the LPS group, the treatment groups with 654-1/654-2 (5 mg/kg) showed a significant improvement in MAP (*P* < 0.01). Moreover, compared to the LPS group, the 654-1/654-2 groups demonstrated a decrease in HR (*P* < 0.01). However, there was no significant difference between 654-1 and 654-2 at the 6-h time point. In conclusion, the administration of 654-1/654-2 resulted in improved hemodynamics. However, it was noted that these levels did not fully normalize when compared to the Con group.

### 3.2 654-1/654-2 inhibited LD, inflammation, and renal dysfunction in septic shock rats

**FIGURE 2:**
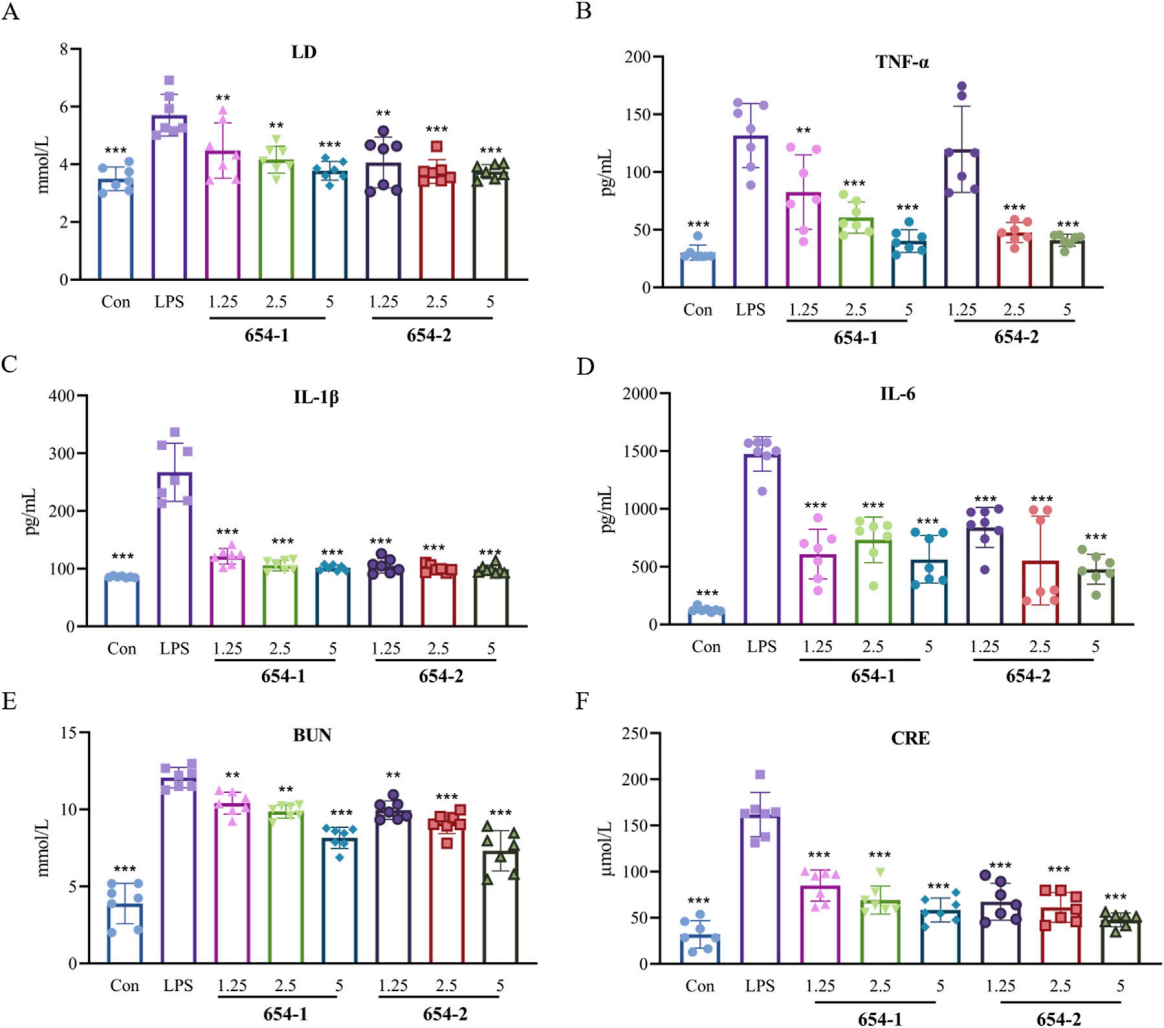
654-1/654-2 improved the levels of lactic acid, inflammation and renal dysfunction markers in plasma in LPS-induced septic shock rats (n = 7) **(A)** LD levels in plasma; **(B)** TNF-α, **(C)** IL-1β and **(D)** IL-6 levels in plasma; **(E)** BUN and **(F)** CRE levels in plasma. Mean ± SD, **P < 0.01, ***P < 0.001 as compared to the LPS group.

As shown in Figure 2, 654-1/654-2 inhibited the upregulation of serum LD expression induced by LPS (Figure 2A). The plasma levels of inflammatory cytokines (Figures 2B–D, IL-1β, IL-6, and TNF-α) and markers of renal dysfunction (Figures 2E, F: CRE and BUN) in the LPS group were significantly elevated compared to the Con group (*P* < 0.001). Except for the 654-2 (1.25) group about TNF-α, the plasma levels of IL-1β, IL-6, TNF-α, BUN, CRE, and LD in the 654-1/654-2 groups (1.25, 2.5 and 5 mg/kg) were significantly or extremely significantly lower than those in the LPS group (*P* < 0.001 or *P* < 0.01). No significant difference was observed between 654-1 and 654-2 groups. Overall, compared to the LPS group, the 654-1/654-2 groups exhibited a dose-dependent decrease in inflammatory cytokines and renal function markers.

### 3.3 654-1/654-2 alleviated kidney injuries in septic shock rats

**FIGURE 3:**
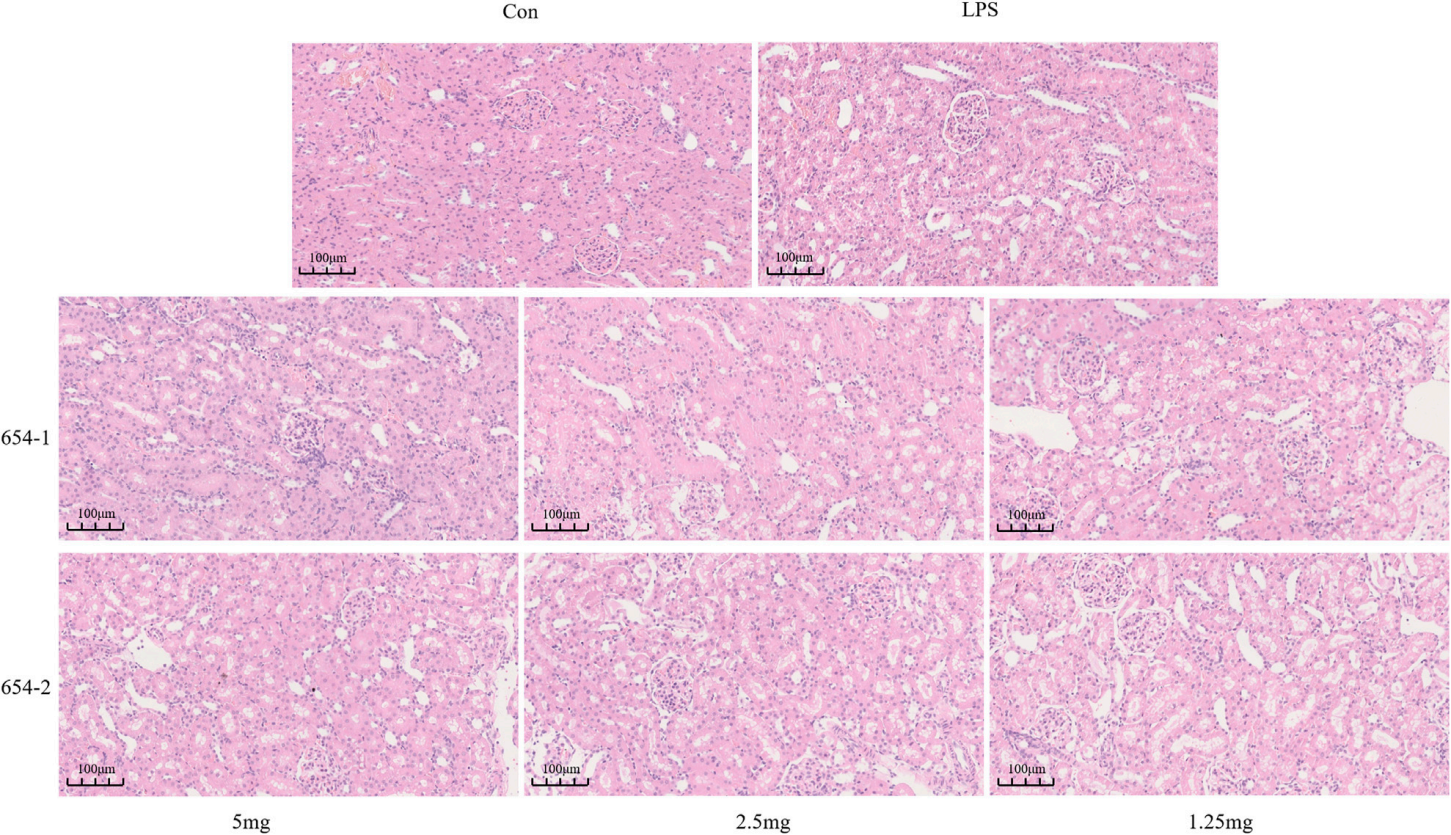
654-1/654-2 ameliorated the H&E staining results of kidney tissue in septic AKI rats (20 × magnification).

Compared to the Con group, the LPS group demonstrated vacuolar degeneration and necrosis of renal tubular epithelial cells, dilation of tubular lumens, partial detachment of epithelial cells from the basement membrane, and the presence of newly regenerated epithelial cells. These alterations were partially alleviated by treatment with 654-1/654-2 (2.5 and 5 mg/kg). However, the 654-1/654-2 (1.25 mg/kg) group did not ameliorate these injuries (Figure 3).

### 3.4 654-1/654-2 inhibited cellular apoptosis and macrophage population in kidney tissues

**FIGURE 4:**
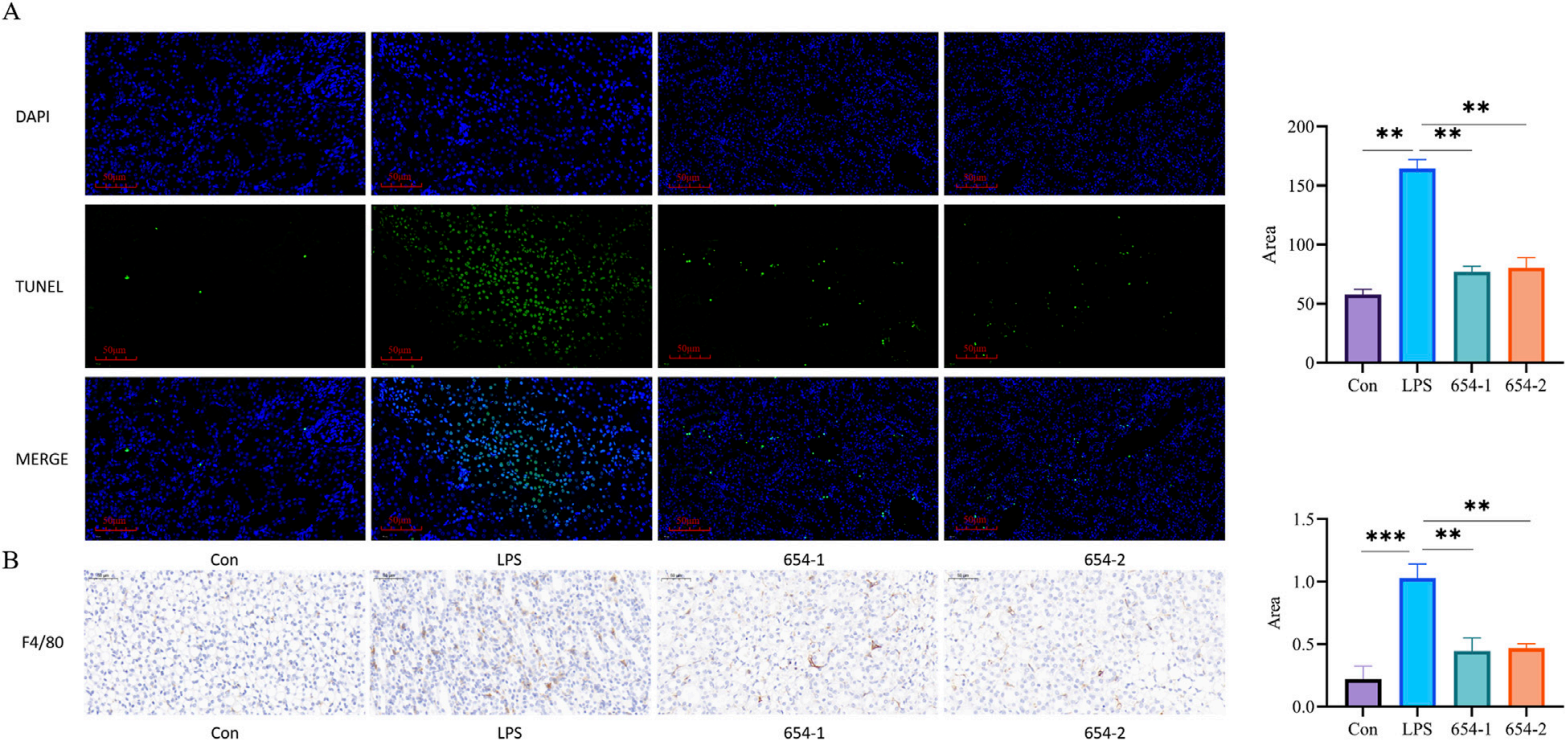
654-1/654-2 (5 mg/kg) reduced apoptosis and macrophage infiltration of kidney tissue in septic AKI rats (n = 3) **(A)** TUNEL staining (20 × magnification. Blue dots indicate normal nuclei and green dots indicate death nuclei.); **(B)** F4/80 IHC staining of kidney tissues (20 × magnification). mean ± SD, **P < 0.01, ***P < 0.001 as compared to the LPS group.

TUNEL assay demonstrated that normal cell nuclei exhibited distinct blue coloration with preserved morphology, while apoptotic cell nuclei appeared green. Compared to the Con group, the LPS group showed a significant increase in TUNEL-positive cells (*P* < 0.001). Treatment with 654-1/654-2 (5 mg/kg) significantly inhibited LPS-induced cellular apoptosis (*P* < 0.01) (Figure 4A). The expression of F4/80 was observed in the cell membrane, with strong positive staining in the LPS group (*P* < 0.001) when compared with the Con group. However, compared with the LPS group, the administration of 654-1/654-2 (5 mg/kg) significantly reduced in F4/80 expression (*P* < 0.01) (Figure 4B).

### 3.5 654-1/654-2 treatment modulated the kidney gene expression pattern

**FIGURE 5:**
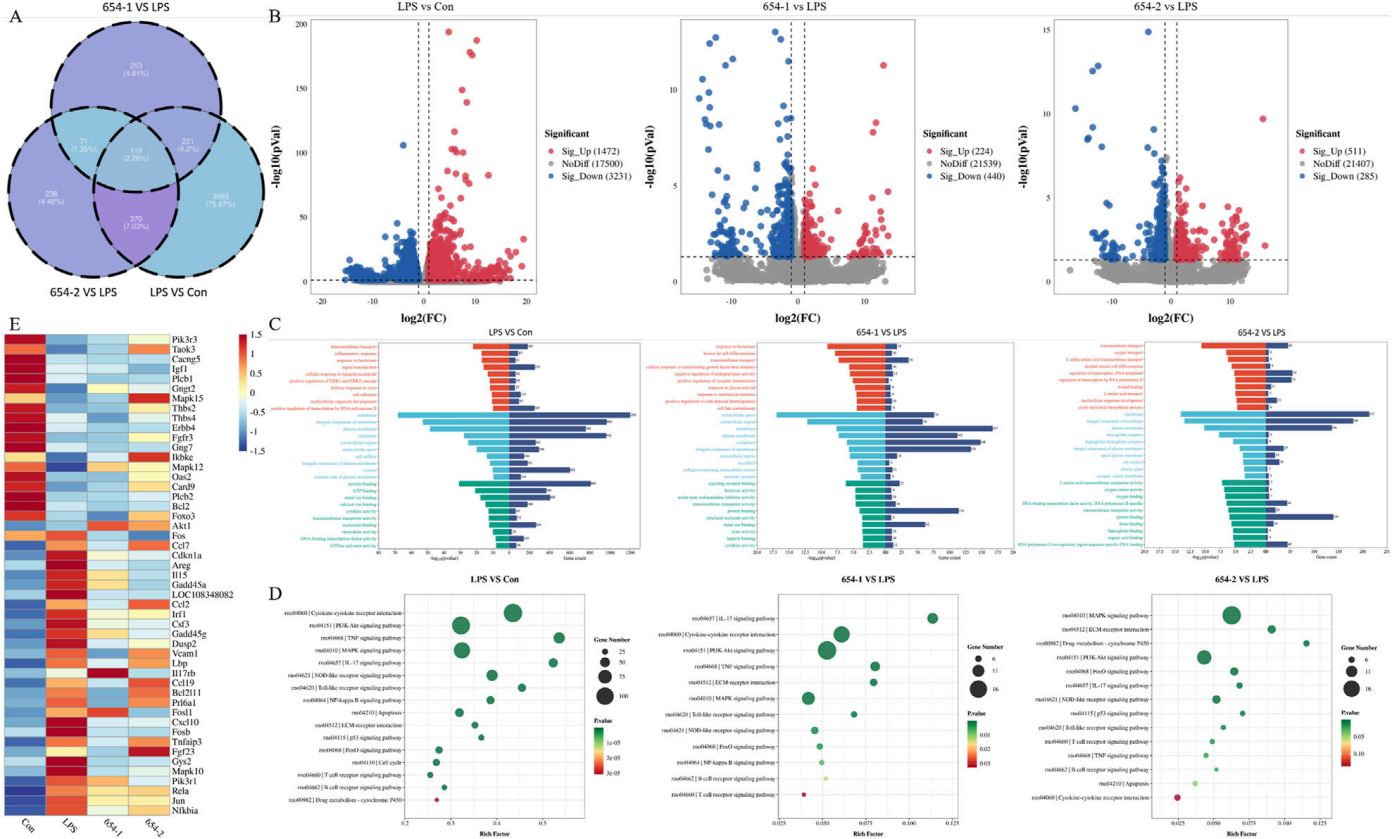
654-1/654-2 (5 mg/kg) ameliorated the renal RNA-seq results in septic AKI rats (n = 3) **(A)** The Venn diagram illustrates the overall gene expression patterns among different comparison groups. **(B)** The volcano plot presents the differential gene expression profiles within each comparison group **(C)** The GO bar chart displays the top ten P-value-ranked biological process (BP), cellular component (CC), and molecular function (MF) information in each comparison group **(D)** The KEGG enrichment bubble plot demonstrates the significantly regulated metabolic pathways associated with septic AKI by LPS, 654-1, and 654-2, respectively. **(E)** The heatmap depicts the overall expression patterns of key significant genes within these pathways across different groups.

According to the Venn diagram (Figure 5A), there are 119 genes present in all groups, while the LPS vs. Con, LPS vs. 654-1, and LPS vs. 654-2 groups have unique genes of 3,993, 253, and 236, respectively. According to the volcano plot (Figure 5B), In the three comparison groups of LPS vs. Con, 654-1 vs. LPS, and 654-2 vs. LPS, 1472,224,511 genes were significantly upregulated and 3,231, 440, 285 genes were significantly downregulated, respectively. GO analysis revealed that 654-1/654-2 primarily regulated transmembrane transport, inflammatory response, and RNA polymerase II transcription in the cell membrane and cytoplasm (Figure 5C). KEGG enrichment analysis indicated enrichment in categories such as metabolism, inflammation, apoptosis, and cytokine receptor interaction. In the comparisons of LPS vs. Con group, 654-1 vs. LPS group, and 654-2 vs. LPS group, enriched signaling pathways included the Pi3k/Akt pathway, MAPK pathway, TNF pathway, Foxo pathway, and IL-17 pathway. The NF-κB pathway was specifically modulated by 654-1, but not regulated by 654-2. Conversely, the apoptotic pathway and P53 signaling pathway were specifically modulated by 654-2, without being influenced by 654-1. (Figure 5D). Based on disease-related pathways, a heat map was generated to visualize the expression patterns of significantly regulated genes, as well as key genes within the pathways, in both the 654-1 vs. LPS and LPS vs. Con comparison groups, or both the 654-2 vs. LPS and LPS vs. Con comparison groups (Figure 5E). Compared to the Con group, LPS significantly downregulated the expressions of genes such as Pik3r3, Mapk12, and Foxo3, and significantly upregulated genes such as Cdkn1a, Mapk10, and Nfkbia. Compared to LPS, 654-1 and 654-2 significantly upregulated the expressions of Mapk12 and Foxo3. Meanwhile, 654-1 and 654-2 significantly downregulated the expression of genes including Cdkn1a, Mapk10, Rela, and Nfkbia when compared to LPS. In summary, these findings suggest that the mechanisms by which 654-1/654-2 alleviate septic shock rat kidney injury are largely similar.

### 3.6 The expressions of 654-1/654-2 on pathways related to inflammation

#### 3.6.1 mRNA expressions of 654-1/654-2 on NF-κB and p38 MAPK pathways

**FIGURE 6:**
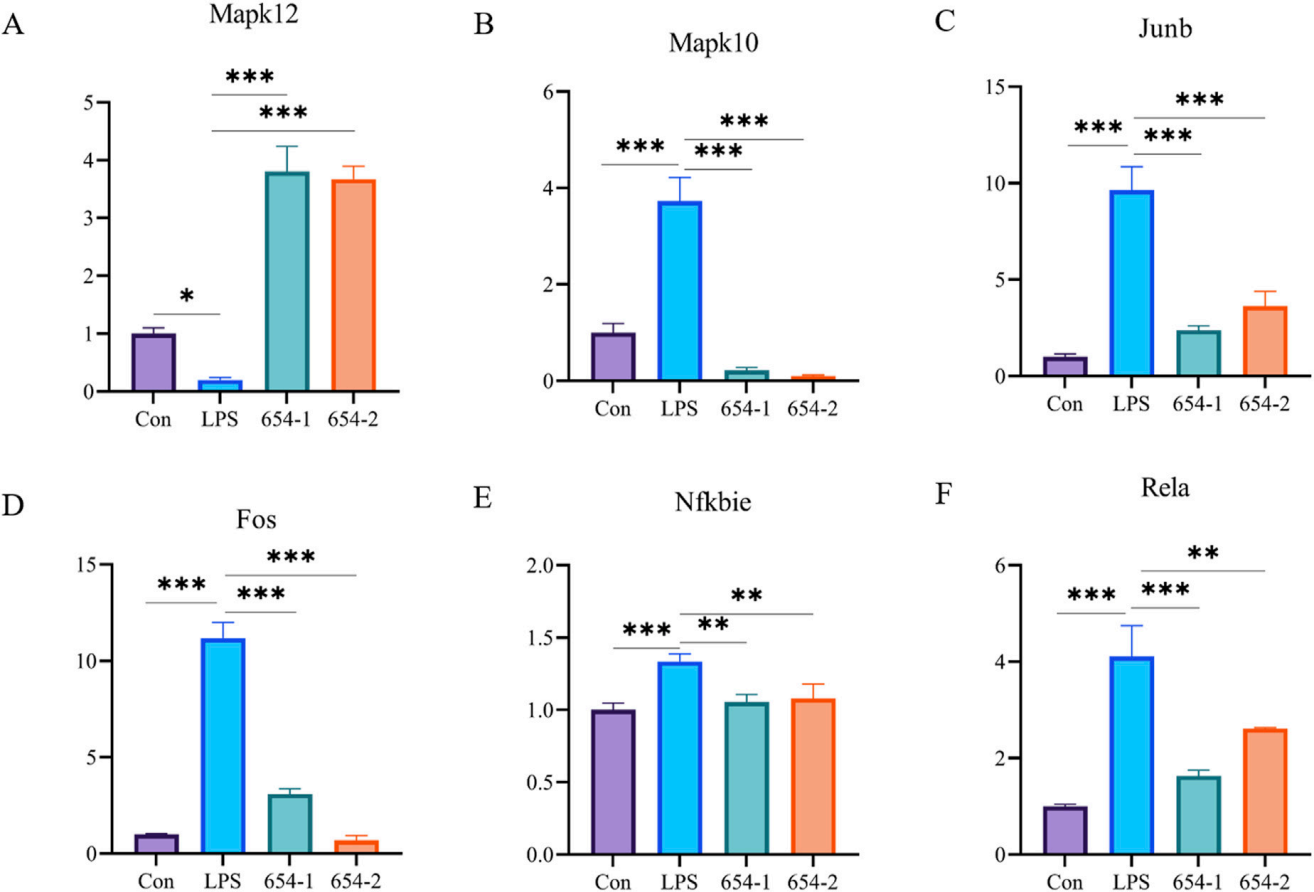
654-1/654-2 (5 mg/kg) ameliorated gene expressions of NF-κB and MAPK pathways in kidney tissues of septic AKI rats. Mean ± SD. *P < 0.05, **P < 0.01, and ***P < 0.01 as compared to the LPS group. **(A–D)** are MAPK pathway-related genes. **(E,F)** are NF-κB pathway-related genes.

To validate the RNA-seq findings, we performed RT-PCR analysis on the core differentially expressed genes within the aforementioned pathways. Compared to the Con group, in the MAPK pathway, the mRNA expression of Mapk12 was significantly downregulated by LPS (*P* < 0.05) (Figure 6A), while the expression of Mapk10, Fos, and Junb was significantly upregulated by LPS, but these effects were significantly reversed by 654-1/654-2 (*P* < 0.001) (Figures 6B–D). In the NF-κB pathway, the mRNA expression of Rela and Nfkbia were significantly upregulated by LPS, but reversed by 654-1/654-2 (*P* < 0.01 or *P* < 0.001) (Figures 6E, F). Interestingly, this result appears to be inconsistent with the RNA-seq enrichment pathway results, in which only 654-1 significantly regulated the NF-κB pathway. However, further analysis of gene expression in RNA-seq revealed that both 654-1 and 654-2 downregulated the expression of Rela and Nfkbia, but not to the same extent, which seems to be the reason for the inconsistent results (Figure 5E).

#### 3.6.2 The protein expression of 654-1/654-2 on NF-κB and p38 MAPK pathways

**FIGURE 7:**
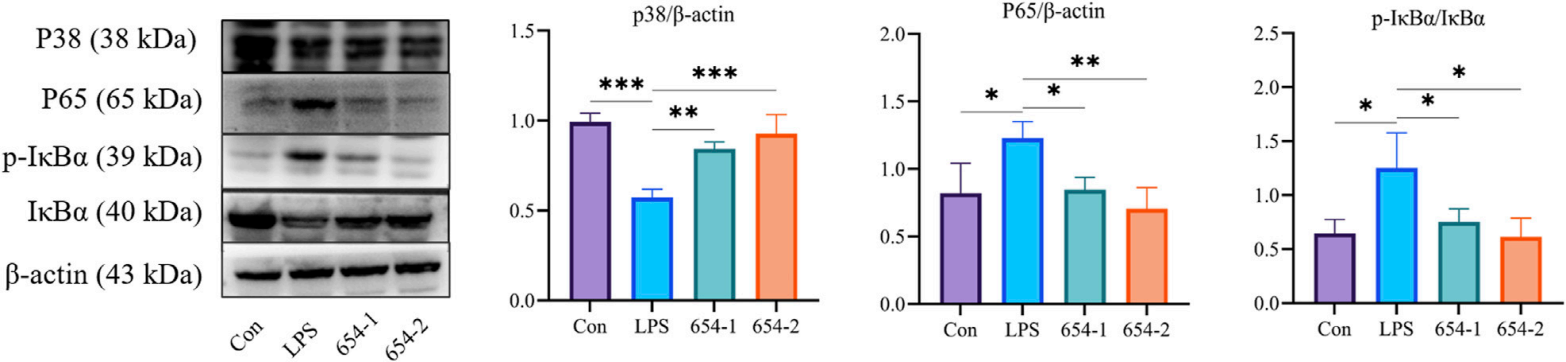
654-1/654-2 (5 mg/kg) ameliorated protein expressions of NF-κB and p38 MAPK pathways in kidney tissues of septic AKI rats. Mean ± SD, *P < 0.05, **P < 0.01, and ***P < 0.01 as compared to the LPS group.

Subsequently, the Western blotting (WB) technique was employed to assess the protein expression levels of the relevant pathways in the kidney. LPS significantly upregulated the protein expression of P65 and p-IκBα (*P* < 0.05), which were reversed by 654-1/654-2 (*P* < 0.05 or *P* < 0.01) (Figure 7). LPS significantly downregulated the protein expression of p38 MAPK (*P* < 0.001), whereas 654-1/654-2 significantly upregulated the protein expression of p38 MAPK (*P* < 0.01 or *P* < 0.001).

### 3.7 654-1/654-2 regulated the apoptosis in kidney tissues

#### 3.7.1 mRNA expressions of 654-1/654-2 on Pi3K/Akt and P53 pathways

**FIGURE 8:**
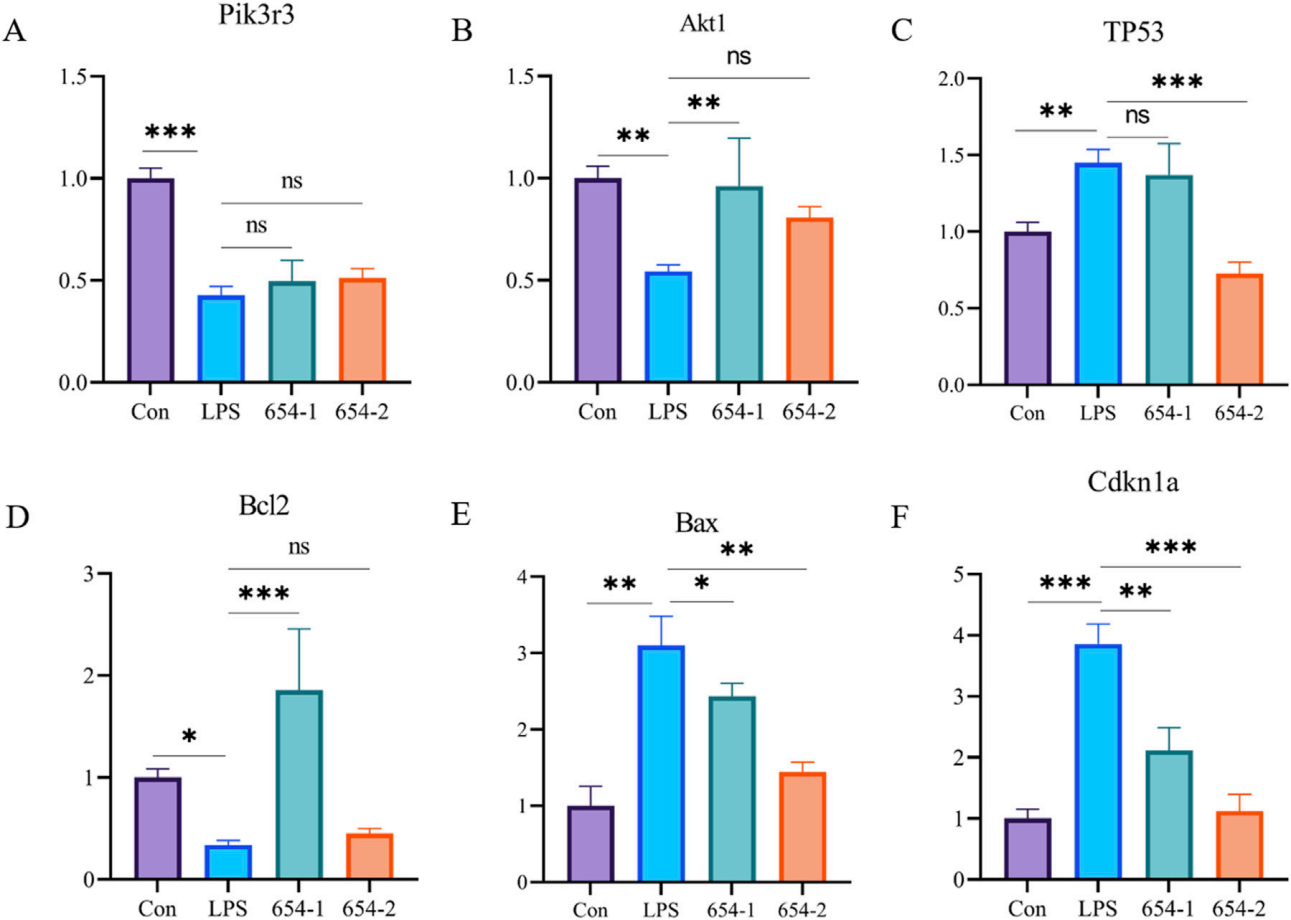
654-1/654-2 (5 mg/kg) ameliorated gene expressions of Pi3K/Akt and P53 pathways in kidney tissues of septic AKI rats. Mean ± SD. *P < 0.05, **P < 0.01, and ***P < 0.001 as compared to the LPS group. **(A)** Pik3r3. **(B)** Akt1. **(C)** TP53. **(D)** Bcl2. **(E)** Bax. **(F)** Cdkn1a.

As shown in Figure 6, compared to the Con group, the mRNA expression of Pik3r3, Akt1, and Bcl2 were significantly downregulated by LPS, and the mRNA expression of TP53, Bax, and Cdkn1a was significantly upregulated by LPS. The mRNA expressions of Bax and Cdkn1a were significantly reversed by both drugs (Figures 8E, F). 654-1 significantly upregulated the mRNA expression of Bcl2, but 654-2 did not exhibit a regulatory effect on Bcl2 (Figure 8D). Additionally only 654-2 significantly downregulated the mRNA expression of P53 (Figure 8C). Notably, 654-1 upregulated the expression of Akt1 significantly and slightly upregulated that of Pik3r3 without significance. Compared with Mod, 654-2 upregulated the expressions of Akt1 and Pik3r3 slightly without any significance, suggesting 654-1, rather than 654-2, activated the Pi3K/Akt pathway (Figures 8A, B). Notably, there was upregulating tendency between LPS and 654-1/654-2 groups, which was partly in consistency with the result of the RNA-seq findings.

#### 3.7.2 Protein expressions of 654-1/654-2 on Pi3K/Akt and P53 pathways

**FIGURE 9:**
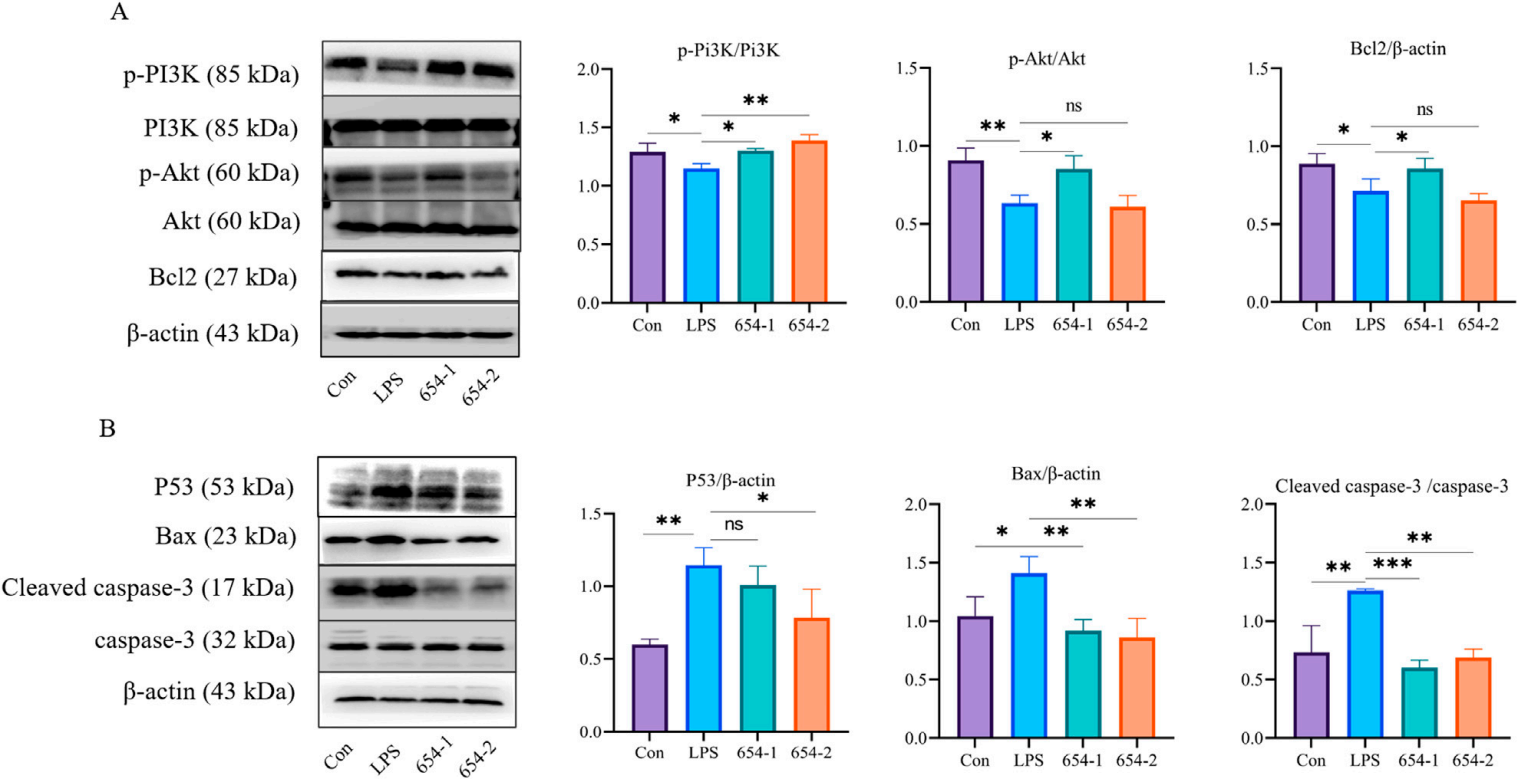
654-1/654-2 (5 mg/kg) ameliorated protein expressions of Pi3K/Akt and P53 pathways in kidney tissues of septic AKI rats **(A)** p-Pi3K, Pi3K, p-Akt, Akt, and Bcl2 protein. **(B)** P53, Bax, cleaved caspase-3/caspase-3 protein. Mean ± SD, *P < 0.05, **P < 0.01, and ***P < 0.001 as compared to the LPS group.

Compared to the control group, LPS significantly downregulated the proteins expression of p-Pi3K, p-Akt, and Bcl2, 654-1 upregulated the proteins expression of Akt1 and Bcl2, but 654-2 did not exhibit regulatory effect on the aforementioned proteins (Figure 9A). LPS significantly upregulated the protein expression of P53, Bax, and cleaved caspase-3 (Figure 9B). 654-2 showed a significant downregulation of P53 protein expression, while 654-1 did not affect its protein expression. 654-1/654-2 significantly reversed the expression of the Bax and cleaved caspase-3 proteins.

## 4 Discussion

Sepsis is essentially a systemic inflammatory response syndrome triggered by infection, resulting in life-threatening organ dysfunction, often including acute kidney injury (Singer et al., 2016). Inflammation and cellular apoptosis are regarded as crucial mediators of organ dysfunction induced by sepsis (Cecconi et al., 2018).

In clinical practice, a decrease in MAP and an increase in LD were key indicators used to identify patients with septic shock (Singer et al., 2016). The inflammatory response induced by LPS leads to the release of significant amounts of IL-1β, IL-6, and TNF-α. These inflammatory cytokines contributed to renal tubular epithelial cell injury, triggering septic AKI (Maremonti et al., 2022). In our study, we observed a significant upregulation of MAP and a downregulation of LDH levels by 654-1/654-2. Furthermore, we observed a reversal of plasma levels of IL-1β, IL-6, and TNF-α, as well as renal function markers BUN and CRE. Additionally, it mitigated LPS-induced renal tubular epithelial cell necrosis and reduced the number of apoptotic cells. These findings indicated that 654-1/654-2 could effectively ameliorate LPS-induced septic AKI.

Next, we employed RNA-seq to elucidate the mechanisms underlying the beneficial effects of 654-1/654-2 in improving septic AKI in rats, as well as the similarities and differences in their mechanisms of action. Specifically, 654-1/654-2 were found to improve septic AKI by modulating pathways including Pi3k/Akt pathway and MAPK pathway. In a rat model of LPS-induced septic AKI, modulation of the MAPK pathway had been shown to inhibit inflammation (Jin et al., 2023). RNA-seq analysis revealed that LPS significantly downregulated RNA expression of p38 MAPK and upregulated expressions of JNK, Fos, and Junb, which were reversed by 654-1/654-2. The seemingly contradictory expression pattern of p38 and JNK could be explained by the fact that specific inhibition of p38 MAPK could activate JNK expression (Muniyappa and Das, 2008; Yue and López, 2020). Fos and Junb were among the structural components of AP-1, and the activity of AP-1 was regulated by JNK (Bejjani et al., 2019). AP-1 serves as a potential regulatory factor for both renal inflammation and cell apoptosis (Shaulian and Karin, 2001; Yu et al., 2023). AP-1-mediated regulation of renal inflammation may occur through the NF-κB pathway (Ruiz-Ortega et al., 2001). 654-1/654-2 inhibited the expression of the NF-κB pathway, as evident from RT-PCR and WB results, thereby suppressing inflammation. Collectively, the above-mentioned studies indicated that 654-1/654-2 upregulated p38 MAPK expression inhibited JNK/AP-1 expression, and subsequently suppressed LPS-induced inflammation in septic AKI rats by downregulating NF-κB level, respectively.

The study highlighted the significant involvement of the Pi3k/Akt pathway in LPS-induced acute kidney injury (AKI). Activation of Pi3k triggered the translocation of Akt to the plasma membrane, leading to its activation. The activation of Akt plays a pivotal role in regulating the expression of key apoptotic proteins, specifically Bcl-2, Bax, and caspase-3 (Zhang et al., 2022). Bcl-2 functions by inhibiting Bax activity, which in turn leads to a reduction in mitochondrial membrane permeability. This inhibition prevents the activation of caspase-3 and ultimately rescues the cells from undergoing apoptosis (Huang et al., 2023). In our investigation, 654-1 treatment upregulated Akt and Bcl-2 expression and downregulated Bax and caspase-3 expressions in RT-PCR and WB, thus mitigating LPS-induced apoptosis in renal tubular epithelial cells.

In contrast, it was observed that 654-2 directly downregulated the expression of Bax and caspase-3, independent of p-Akt and Bcl-2 regulation. This suggests that 654-2 may potentially regulate apoptosis through alternative pathways. Previous studies have indicated that phosphorylated P53 induced apoptosis by promoting Bax expression (Follis et al., 2015). Our RNA-seq data revealed that only 654-2 exhibited regulation of LPS-induced apoptosis through the P53 pathway, whereas 654-1 had no impact on the P53 pathway. Both WB and RT-PCR analyses confirmed that only 654-2 regulated the mRNA and protein expression of P53, whereas 654-1 did not affect its expression. These findings suggest that 654-2 may modulate apoptosis in renal tubular epithelial cells via the P53 pathway. In conclusion, both 654-1 and 654-2 inhibited LPS-induced apoptosis in renal tubular epithelial cells; however, their mechanisms of action were different. Specifically, 654-1 inhibited apoptosis through the Pi3K/Akt/Bcl-2 pathway, whereas 654-2 inhibited apoptosis via the P53/Bax pathway.

## 5 Conclusion

In conclusion, our findings demonstrated that 654-1/654-2 played a crucial role in protecting against septic AKI through their anti-inflammatory and anti-apoptotic effects. The joint upregulation of p38 MAPK by 654-1/654-2 led to the suppression of JNK/AP-1 expression, resulting in the downregulation of NF-κB levels, thereby inhibiting inflammation in septic AKI rats. However, the mechanisms of action differ between the two compounds. Specifically, 654-1 inhibited cell apoptosis via the Pi3k/Akt/Bcl-2 pathway, whereas 654-2 exerted its anti-apoptotic effects through the P53/Bax pathway. These findings provided strong support for the utilization of 654-1/654-2 in clinical practice for the treatment of organ damage caused by septic shock.

## Data Availability

The datasets presented in this study can be found in online repositories. The names of the repository/repositories and accession number(s) can be found below: https://www.ncbi.nlm.nih.gov//bioproject/PRJNA1127205.
