# Supplementary material for: Anisodamine (654-1/654-2) ameliorates septic kidney injury in rats by inhibiting inflammation and apoptosis
Source: Front Pharmacol. 2024 Sep 27;15:1421551. doi: 10.3389/fphar.2024.1421551 (PMC11467892; doi:10.3389/fphar.2024.1421551)
Supplement: Supplementary file 1 [file DataSheet1.PDF]

Supplementary

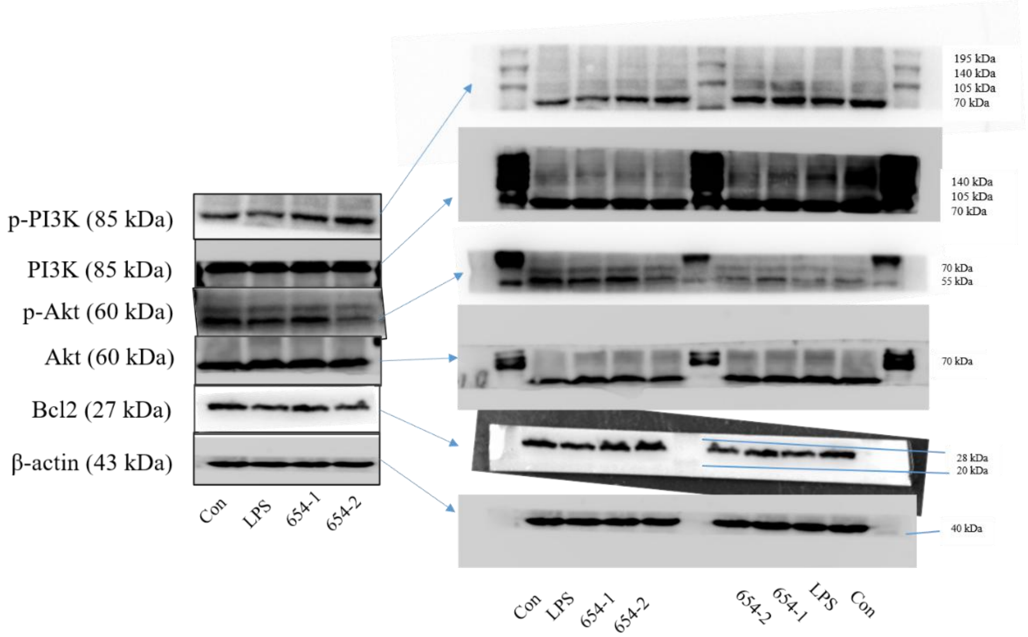

Supplementary Figure S1. The blots were cut prior to detection with antibodies during blotting.

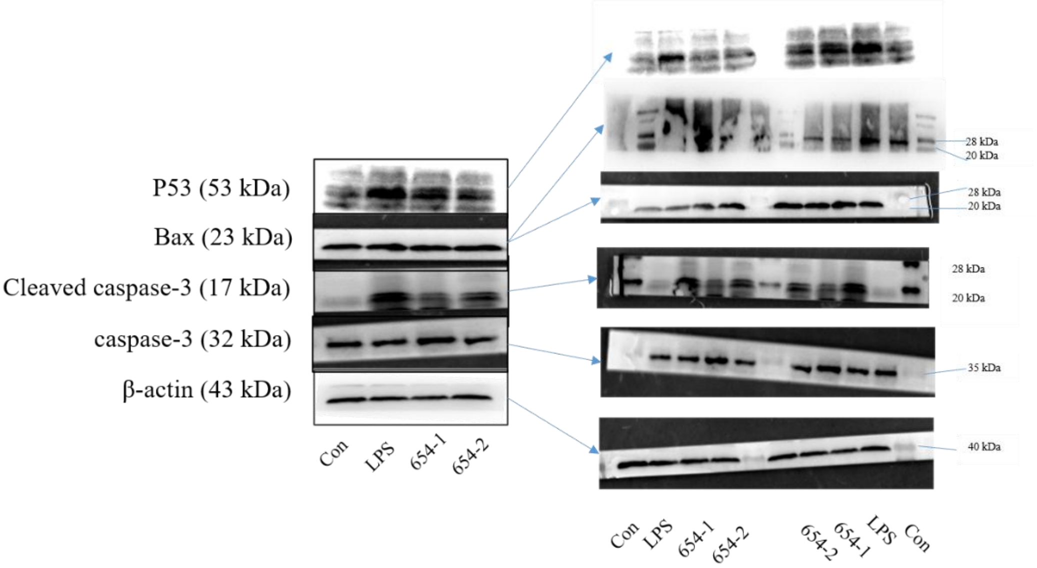

Supplementary Figure S2. The blots were cut prior to detection with antibodies during blotting.

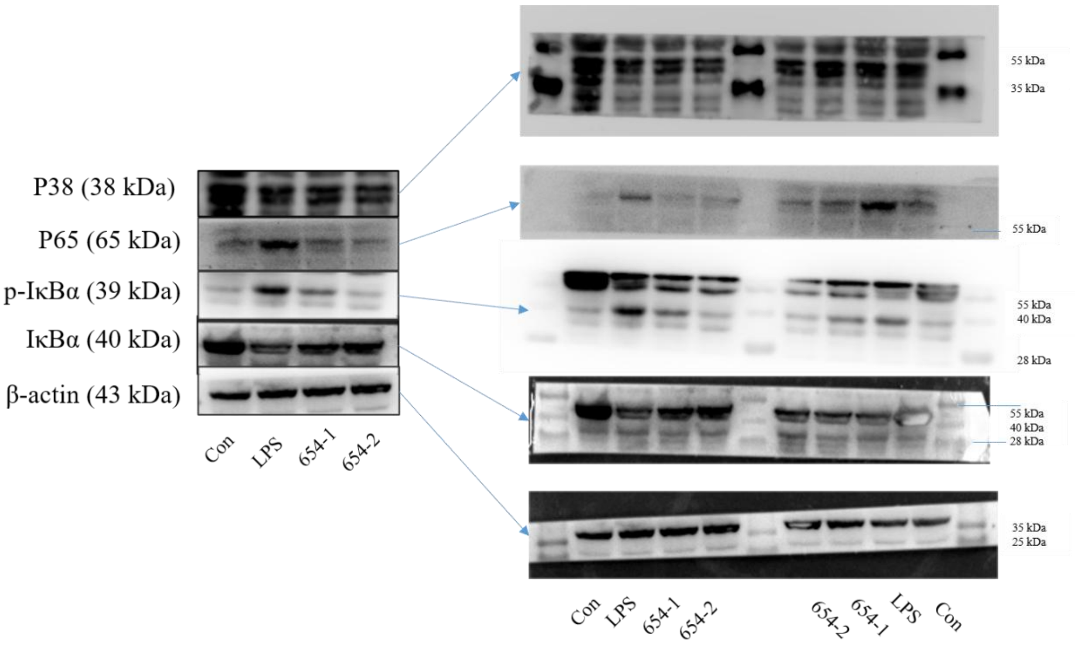

Supplementary Figure S3. The blots were cut prior to detection with antibodies during blotting.
